# Functional expression of a penicillin acylase from the extreme thermophile *Thermus thermophilus* HB27 in *Escherichia coli*

**DOI:** 10.1186/1475-2859-11-105

**Published:** 2012-08-09

**Authors:** Leticia L Torres, Eloy R Ferreras, Ángel Cantero, Aurelio Hidalgo, José Berenguer

**Affiliations:** 1 Centro de Biología Molecular “Severo Ochoa” (UAM-CSIC), Nicolás Cabrera 1, 28049, Madrid, Spain

**Keywords:** Penicillin acylase, *Thermus thermophilus*, Pre-pro-protein, Autoprocessing, Thermophile, Thermozyme

## Abstract

**Background:**

Penicillin acylases (PACs) are enzymes of industrial relevance in the manufacture of β-lactam antibiotics. Development of a PAC with a longer half-life under the reaction conditions used is essential for the improvement of the operational stability of the process. A gene encoding a homologue to *Escherichia coli* PAC was found in the genome of the thermophilic bacterium *Thermus thermophilus* (*Tth*) HB27. Because of the nature of this PAC and its complex maturation that is crucial to reach its functional heterodimeric final conformation, the overexpression of this enzyme in a heterologous mesophilic host was a challenge. Here we describe the purification and characterization of the PAC protein from *Tth* HB27 overexpressed in *Escherichia coli.*

**Results:**

Fusions to a superfolder green fluorescent protein and differential membrane solubilization assays indicated that the native enzyme remains attached through its amino-terminal end to the outer side of the cytoplasmic membrane of *Tth* cells. In order to overexpress this PAC in *E. coli* cells, a variant of the protein devoid of its membrane anchoring segment was constructed. The effect of the co-expression of chaperones and calcium supplementation of the culture medium was investigated. The total production of PAC was enhanced by the presence of DnaK/J and GrpE and even more by trigger factor and GroEL/ES. In addition, 10 mM calcium markedly improved both PAC specific and volumetric activities. Recombinant PAC was affinity-purified and proper maturation of the protein was confirmed by SDS-PAGE and MALDI-TOF analysis of the subunits. The recombinant protein was tested for activity towards several penicillins, cephalosporins and homoserine lactones. Hydrophobic acyl-chain penicillins were preferred over the rest of the substrates. Penicillin K (octanoyl penicillin) was the best substrate, with the highest specificity constant value (16.12 mM^-1^.seg^-1^). The optimum pH was aprox. 4 and the optimum temperature was 75 °C. The half-life of the enzyme at this temperature was 9.2 h.

**Conclusions:**

This is the first report concerning the heterologous expression of a *pac* gene from a thermophilic microorganism in the mesophilic host *E. coli*. The recombinant protein was identified as a penicillin K-deacylating thermozyme.

## Background

Penicillin acylase (PAC, EC 3.5.1.11) is one of the most relevant enzymes in the pharmaceutical industry. It is used in the production of 6-amino penicillanic acid (6-APA), which is subsequently used in the chemical synthesis of new lactams with greater effectiveness. PACs belong to the N-terminal nucleophile hydrolase family, whose members undergo a complex maturation process. In this process, the pre-pro-protein is synthesized and translocated to the periplasm with the concomitant removal of the signal peptide. The resulting pro-protein is then autoproteolyzed in the periplasm rendering the β-subunit. A second autoproteolysis detaches the α-subunit and uncovers the active site by elimination of a spacer peptide [1]. The precision of the maturation process is extremely relevant for the functionality of the final heterodimeric protein (α- plus β-subunits).

Industrially, the penicillin G acylase (PGA) from *Escherichia coli* is the enzyme of choice, whether recombinant or native. Although the optimum temperature for the hydrolysis of penicillin G is 50°C, the enzyme loses stability above 30°C and must be used in immobilized form. Other described PGAs with higher stability are those from *Alcaligenes faecalis* (AfaePGA, *t*_1/2_ of 15 min at 55°C), *Bacillus badius* (*t*_1/2_ of 20 min at 55°C) and *Achromobacter xylosoxidans* (*t*_1/2_ of 55 min at 55°C). Their thermostability arises from different reasons such as additional disulfide bonds, more salt bridges or additional buried ionic pairs, respectively [2,3]. However, operational stability of the catalytic process could be strongly improved by the use of enzymes with a longer half-life under the reaction conditions regularly used.

Bearing in mind the above-mentioned limitations of penicillin acylases and the naturally superior operational stability of thermozymes, a putative *pac* gene (NCBI_accession number TTC1972) was identified in the genome of the thermophilic bacterium *Thermus thermophilus* (*Tth*) HB27, a microorganism with an optimum growth temperature of 75°C [4]. Enzymes isolated from extreme thermophiles (thermozymes) show an optimum temperature similar to that of maximum growth rate of its source. In addition, thermozymes exhibit an above-average resistance to chemical denaturation, for instance, caused by organic solvents, detergents or pH. Thus, the *Tth*PAC protein would be an extremely stable catalyst suitable for the industrial production of semi-synthetic penicillins. Generally speaking, fermentation of the natural thermophilic host is not economically viable due to the nutritional and energy requirements of the process. Thus, expression of the protein of interest in a recombinant system is usually preferred. However, recombinant expression of proteins from thermophiles in mesophiles is frequently a challenge. In fact, it is estimated that less than 20% of the ORFs of any extreme thermophile or hyperthermophile genome may be expressed and folded correctly in *E. coli*[5]. Strategies to improve folding of thermozymes expressed recombinantly in *E. coli* include growth of the host at higher temperature [6], refolding of the denatured protein in the presence of cofactors, or co-expression with chaperones from *E. coli*[7].

In this work, we report the first characterization of a penicillin acylase from a thermophile. With the objective of a potential industrial application in mind:

we performed differential fractionation of the TthPAC combined with detergent solubility and trypsin accessibility assays and characterized the enzyme as a periplasmic, membrane-bound heterodimer in its native host, which makes TthPAC overproduction in *Thermus* impractical.

TthPAC was expressed recombinantly in the cytoplasm of *E. coli* devoid of its signal peptide but nevertheless, the existence of autoproteolytic maturation leading to a functional enzyme was achieved.

we improved productivity and activity of TthPAC in *E. coli* by coexpression with chaperones and Ca^2+^ supplementation, respectively.

finally, we characterized the enzyme with different amides and determined the enzyme to be most proficient in the hydrolysis of penicillin K, similarly to the acylases of *Streptomyces lividans* and *Actinoplanes utahensis*.

## Results and discussion

### *Tth*PAC is produced and processed in *T. thermophilus*

**Figure 1:**
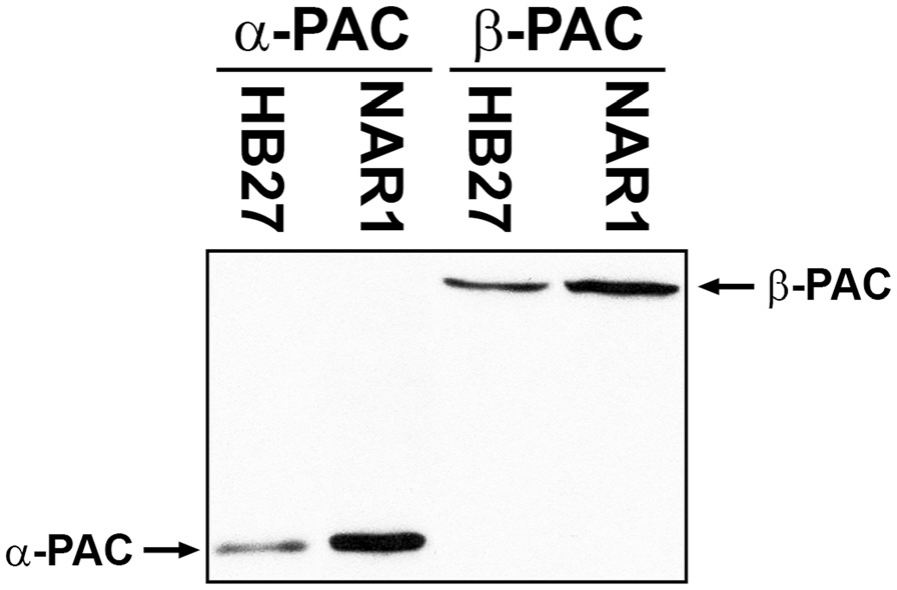
**Presence of PAC protein in* Tth *cells.** Total proteins from * Tth *cells of HB27 and NAR1 strains were separated by SDS-PAGE and the presence of α- and β-PAC subunits was determined individually by western blot assays.

In the first place and in order to be able to study *Tth*PAC production and maturation, we generated antibodies against the α- and β-subunits of the PAC codified in the *Tth* HB27 genome, separately produced in *E. coli* cells. Each antiserum recognized only the corresponding subunit of the PACs from the HB27 and NAR1 *Tth* strains (Figure 1), demonstrating: (i) their specificity, (ii) the presence of constitutively expressed PAC protein in these strains, and (iii) the existence of a maturation process of the pro-PAC into two subunits, similar to the one described for mesophilic PACs [8,9]. The apparent electrophoretic mobilities of these protein subunits are 22 and 60 kDa for the α- and β-subunits, respectively.

### Subcellular localization of * Tth *PAC

**Figure 2:**
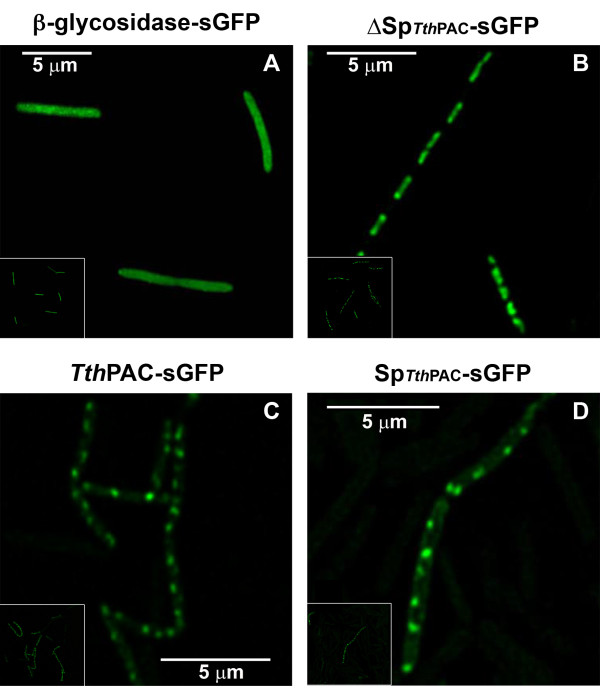
*** Tth *PAC subcellular location.** Fluorescence confocal microscopy images were taken from exponential cultures of *Tth* HB27 Δ*pac* mutant strain harboring β-glycosidase-sGFP (**A**), ΔSp*Tth*PAC-sGFP (**B**), *Tth*PAC-sGFP (**C**) or Sp_*Tth*PAC_-sGFP (**D**) protein fusions.

PACs are soluble enzymes in mesophilic organisms. They are usually periplasmic proteins in Gram-negative bacteria, secreted to the media in most Gram-positive bacteria, and can even be intracellular proteins in particular genera like * Bacillus *[10,11]. However, there is no report about the final destination of PAC proteins in thermophilic bacteria. Consequently, in order to shed some light on * Tth *PAC subcellular location, fusions of * Tth *PAC to superfolder GFP (sGFP, a variant of the * Aquorea victoria * green fluorescent protein that fluoresces properly at 70°C) [4,12] and membrane differential solubilization assays were performed. The sGFP was fused to the entire * Tth *PAC (* Tth *PAC-sGFP), to a * Tth *PAC devoid of its putative 47-amino acid signal peptide (ΔSp* Tth *PAC-sGFP), to the putative * Tth *PAC signal peptide alone (Sp_*Tth*PAC_-sGFP) and to a β-glycosidase (NCBI accession number YP_006025) used as a cytoplasmic marker (β-glycosidase-sGFP). As expected for a cytoplasmic soluble protein, the β-glycosidase-sGFP fusion resulted in a homogeneous distribution of the fluorescence throughout the bacteria (Figure 2a). In ΔSp* Tth *PAC-sGFP fusions, fluorescence is also located in the cytoplasm but with a defined distribution on central regions of the cells (Figure 2b). When analyzing * Tth *PAC-sGFP or Sp_*Tth*PAC_-sGFP fusions, the observed fluorescence intensity was lower and located at polar/subpolar loci in the envelope of the bacteria, indicating either a membrane or a periplasmic location of the chimeric protein (Figure 2c and 2d).

**Figure 3:**
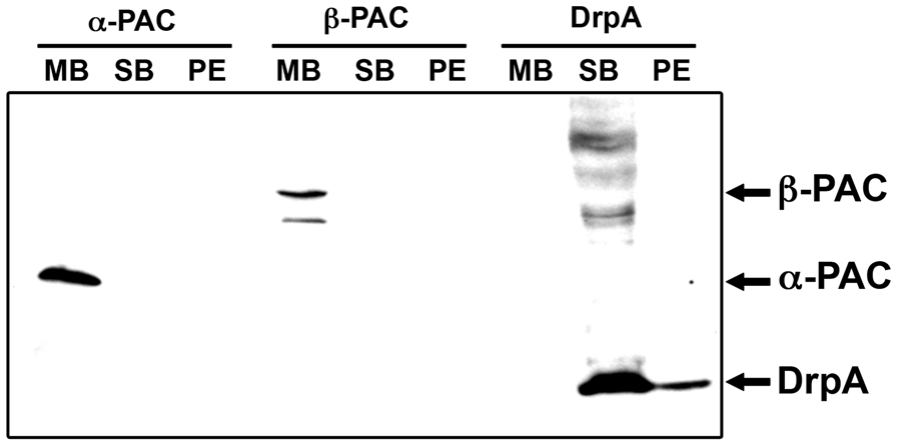
**Immunodetection of* Tth *PAC in the membrane fraction.** The presence of α- and β-PAC subunits and of the periplasmic protein DrpA in the membrane fraction (MB), the soluble fraction (SB) and the periplasmic fraction (PE) of *Tth* NAR1 Δ*slpA* multicellular bodies was analyzed by western blot assays.

In order to determine whether *Tth*PAC is being transported to the periplasmic space, we took advantage of the use of a *Tth* NAR1 mutant in the *slpA* (S-layer protein) gene. This strain forms round multicellular bodies surrounded by a common envelope that facilitates purification of periplasmic proteins [13]. DrpA (a nitrate respiration system regulatory protein) was used as a periplasmic protein marker. Surprisingly, we observed that in contrast to what happens in other Gram-negative bacteria, PAC is not being secreted or soluble at all but remains anchored to the membrane of *Tth* NAR1 Δ*slpA* cells (Figure 3). Note that DrpA is present in the soluble fraction because of the extraction method employed, which cannot deal with undisrupted multicellular bodies or single cells.

**Figure 4:**
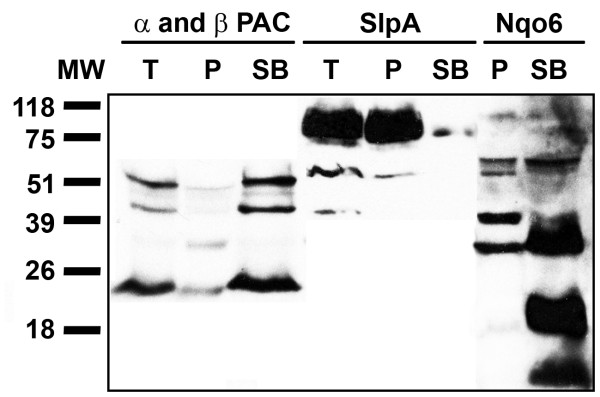
*** Tth *inner membrane solubilization and immunodetection of* Tth *PAC.** PAC was immunodetected in *Tth* HB27 isolated membranes after a 30-min treatment with Sarkosyl at 37°C. T, total membranes; P, insoluble membrane fraction after detergent treatment; SB, fraction of soluble proteins after detergent treatment. SlpA, outer membrane protein marker; Nqo6, inner membrane protein marker.

The use of the mild detergent Sarkosyl has been described as a method to selectively solubilize inner membrane proteins of Gram-negative bacteria [14-17]. PAC and the inner membrane marker Nqo1 became soluble after the detergent treatment, while SlpA, an outer membrane protein, remained insoluble under the same conditions (Figure 4). These data indicate that PAC is anchored to the inner membrane of *Tth* cells.

**Figure 5:**
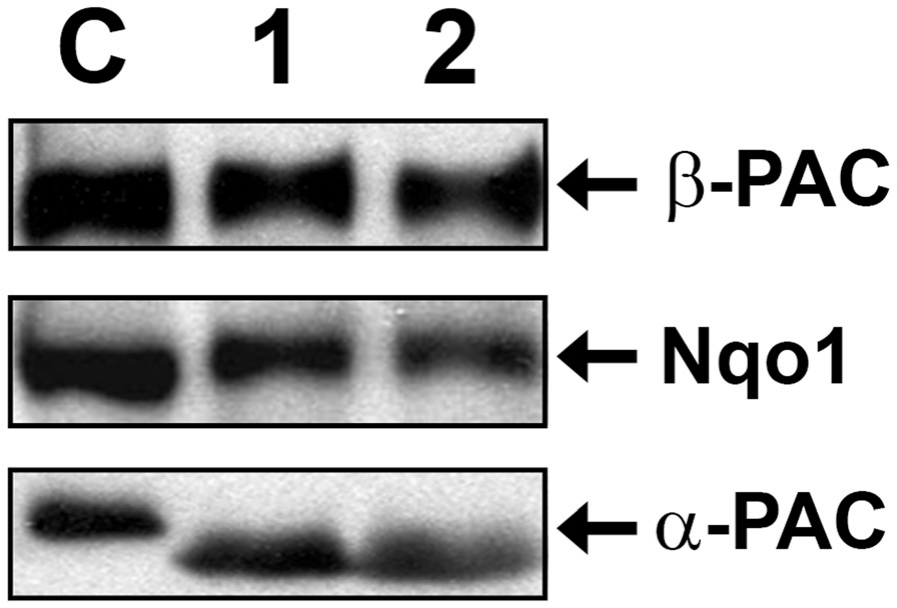
**Trypsin accessibility assays.*** Tth *PAC α- and β-subunits were immunodetected in * Tth * NAR1 Δ* slpA * strain before trypsin treatment (C), and after a digestion with 1.2 mg/ml of trypsin (1) or with 5.5 mg/ml of trypsin (2). EDTA at 5 mM was used to get a partial solubilization of the outer membrane. Nqo1 was used as a cytoplasmic marker.

Whether PAC is oriented towards the cytoplasm or the periplasmic space of *Tth* cells was studied through trypsin accessibility assays. They were carried out in the *Tth* NAR1 Δ*slpA* mutant [13] in order to avoid impediments to the trypsin entrance. Additionally, EDTA was used to partially solubilize the outer membrane. Despite the fact that the *Tth*PAC β-subunit was unaffected, the molecular weight of the α-subunit was reduced after a 1–2 minute trypsin treatment (Figure 5). Immunodetection of Nqo1 under the same conditions showed less accessibility to degradation, suggesting that PAC was oriented towards the periplasmic space.

**Figure 6:**
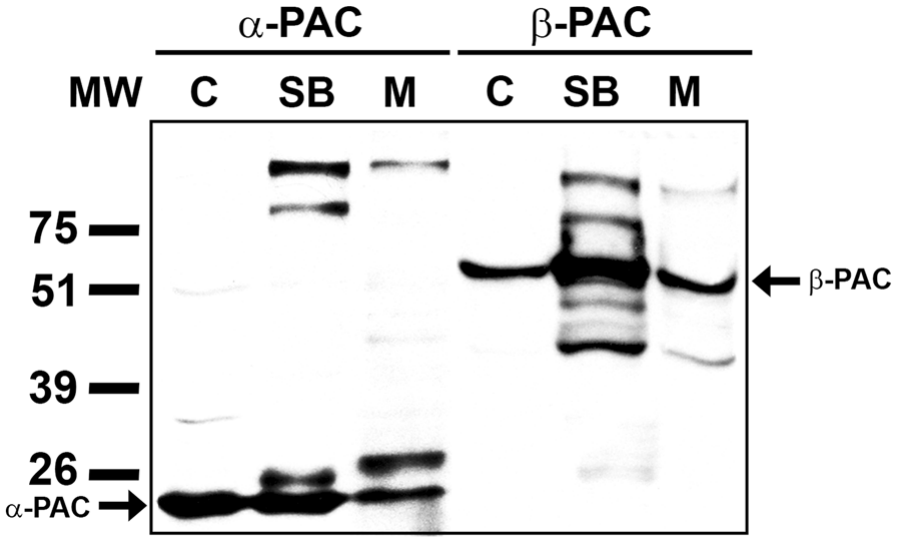
**Subcellular location of the quimeric protein Sp**_*** Eco ***_**-PAC**_*** Tth ***_**in* Tth *cells.** The overexpression was performed in the Δ* pac Tth * mutant strain. Immunodetection of α- and β-PAC subunits was performed on the soluble protein fraction (SB) or in the membrane fraction (MB).* Tth *PAC matured in *Tth* cells was used as control (C).

The high content of hydrophobic amino acids on the N-terminal sequence of * Tth *PAC suggests that this portion of the protein by itself could anchor the enzyme to the inner membrane of the bacteria. To evaluate this, we constructed a chimeric protein using the signal peptide of * E. coli * PGA and the * Tth *PAC protein devoid of the first 32-amino acids from the N-terminal end. The chimeric protein (Sp_*Eco*_-PAC_*Tth*_) was expressed in a *Tth* Δ*pac* mutant mainly as a soluble protein and was processed into α- and β-subunits (Figure 6). These data indicate that the N-terminal sequence of PAC is the responsible for its anchoring to the membrane.

In mesophilic bacteria, PACs have been reported either as cytosolic or periplasmic proteins [10,11]. However, the data presented here show that * Tth *PAC is attached to the external face of the cytoplasmic membrane, thus broadening the possible subcellular locations of the PACs to be studied hereafter. Based on the sGFP fusion experiments, we suggest that *Tth*PAC is being directed to punctual secretion points of the cytosolic membrane, through the twin-arginine system (TAT), that has been shown to form foci near the cell poles in other bacteria [18]. This is because sGFP folds in the cytoplasm [19] and the only secretion system that enables the transport of folded proteins is the TAT system. Also, the presence of two arginines in the N-terminal signal peptide of *Tth*PAC protein supports this idea. In summary, we suggest *Tth*PAC carries a non-cleavable TAT signal peptide that operates as a signal anchor domain. A similar case has been described for a Rieske Fe-S protein, whose TAT signal peptide is not processed but ultimately forms a transmembrane helix through the lipid bilayer [20]. Whether the membrane location of PACs is a feature that could be extended to other thermophilic bacteria is an issue to be elucidated.

### Homologous expression

**Figure 7:**
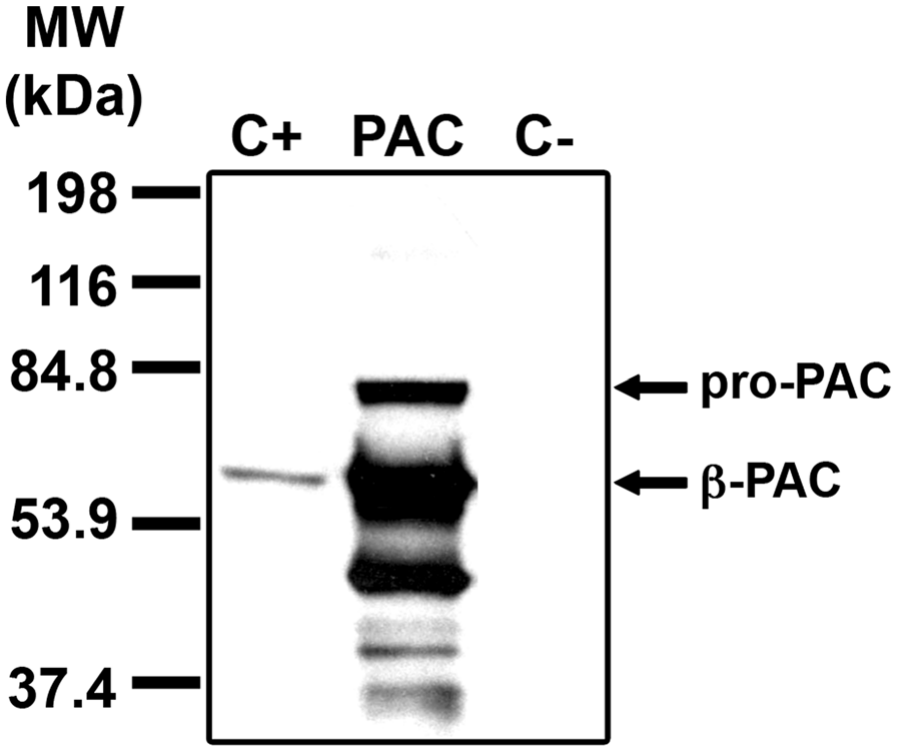
*** Tth *PAC overexpression in* Tth *Δ* pac *cells.***Tth*PAC β-subunit was immunodetected in the * Tth *HB27 wild type strain (C+), in the * Tth *Δ*pac* mutant strain transformed with pWURPAC (PAC) and in the * Tth *Δ*pac* mutant (C-).

Due to the complexity of its processing, we first tried to overexpress the *Tth*PAC protein directly in *Tth* cells. For this the *pac* gene was amplified by PCR, ligated to the pWUR112/77-1 [21] plasmid and overexpressed in a *Tth*Δ*pac* mutant strain. As shown in Figure 7, from the total amount of pre-pro-PAC obtained only a fraction was auto-processed. Attempts to purify this mature * Tth *PAC fraction proved difficult and resulted in low yields. Besides, unprocessed PAC as well as proteolytic polipeptides were found, indicating that the auto-proteolytic processing of the pro-protein is compromised when an overexpression is performed, even in* Tth *cells (Figure 7). Because the efficiency of protein translocation through the TAT system is dependent on the amount of available transporter, the overproduction of * Tth *PAC could have overwhelmed the secretion machinery, thus making the fraction of pre-pro-PAC that remained in the cytoplasm to mature improperly.

### Heterologous expression of * Tthpac * in * E. coli * cells

**Figure 8:**
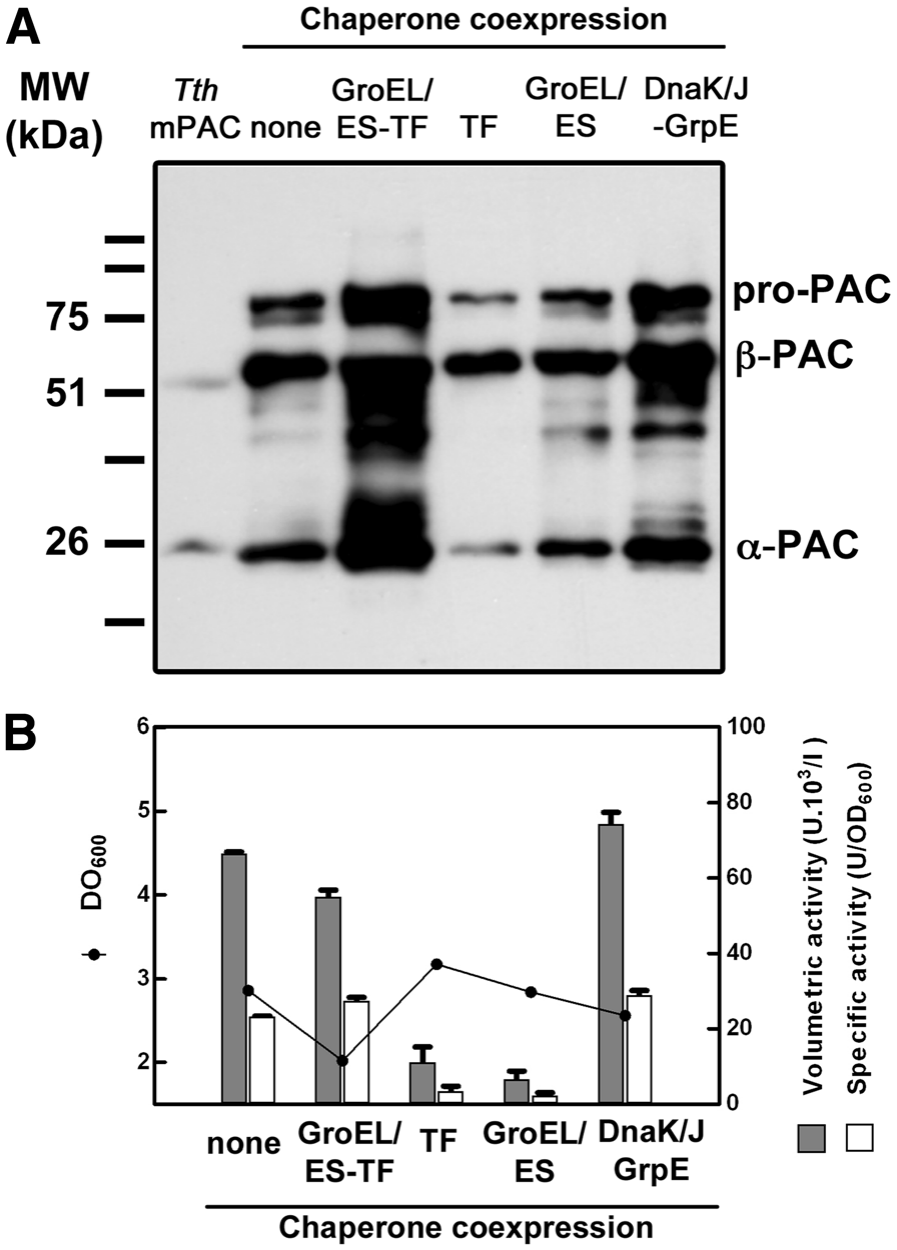
**Effect of chaperone co-expression on* Tth *PAC overproduction in* E. coli *cells.** (**A**) Western blot against α- and β-*Tth*PAC subunits. Lane 1 (C), *Tth*PAC matured in *Tth*; lane 2, *Tth*PAC overexpression without chaperone co-expression; lane 3 to 6, co-expression of *Tth*PAC with GroEL/ES-trigger factor (TF), TF alone, GroEL/ES, and DnaK/J-GrpE, respectively. (**B**) Optical density at 600_nm_ (dots), specific (white bars) and volumetric (grey bars) activity of *Tth*PAC in the absence (none) or presence of chaperones (GroEL/ES-TF, TF alone, GroEL/ES or DnaK/J-GrpE).

Because of the results shown above, we tried to overexpress the *Tth*PAC in *E. coli*. Since the N-terminal end of *Tth*PAC turned out to be membrane anchored, two approaches were undertaken in order to manage *Tth*PAC overexpression in *E. coli* cells. We constructed a 5´shortened version of the *pac* gene (ΔSp-*Tthpac*) and a fusion of the former with the leader sequence of the *E. coli pga* gene (Sp_*Eco*_-*pac*_*Tth*_). The chimeric protein Sp_*Eco*_-PAC_*Tth*_ was efficiently translocated to the periplasmic space of *E. coli*, but the maturation of the protein into α- and β-subunits of the correct size failed [Additional file 1]. As translocation to another cell compartment often proves to be a bottleneck dependent on the efficiency and amount of available transporter, we focused on the expression of ΔSp-*Tth*PAC, likely soluble into the cytoplasm of *E. coli*. Because a proper folding is necessary to achieve a successful pro-protein maturation, the effect of chaperone assistance was studied in the course of PAC overexpression. A selection of cytoplasmic chaperones, including trigger factor (TF), GroEL/ES, and DnaK/J-GrpE (TaKaRa Bio Inc.), were co-expressed with ΔSp-*Tth*PAC. Pro-PAC production and processing was evaluated by western blot, while *Tth*PAC functionality was determined by measuring its enzymatic activity. As shown in Figure 8a, production of pro-PAC was enhanced by the presence of DnaK/J and GrpE and even more by the combination of TF and GroEL/ES. Also, in all conditions, a fraction of pro-PAC was processed into α- and β-subunits that showed electrophoretic migrations similar to the PAC subunits obtained from *Tth* cells, indicating that the post-translational processing steps for *Tth*PAC maturation can occur also in the cytoplasm of *E. coli.* However, when analyzing *Tth*PAC activity in the same conditions we observed that both specific and volumetric activities in the culture without chaperone co-expression were similar to those observed in cultures with DnaK/J and GrpE or TF and GroEL/ES co-expression (Figure 8b). These results indicate that the production of mature *Tth*PAC in *E. coli* cells can be increased through the co-expression of appropriate chaperone(s), but the fraction of *Tth*PAC protein that is enzymatically active cannot be assumed from the total amount of *Tth*PAC that is produced.

The use of cytoplasmic or periplasmic chaperones in the folding assistance of PACs overproduced in heterologous hosts has been widely reported [22-26]. As for *Tth*PAC, TF and GroEL/ES proved to be the best choices at improving *Eco*PGA production [22]. However, in disagreement with the results reported by Xu et al. [22], we found that *Tth*PAC activity in the presence of these chaperones was reduced when compared to the absence of folding modulator co-expression. We hypothesize that *Tth*PAC remains attached to the chaperone after its folding assistance, so a proper *Tth*PAC catalysis is prevented. Chaperone detaching experiments based on the use of ATP/Mg^+2^ incubations were carried out, and a *Tth*PAC release from GroEL/ES complex was observed [Additional file 2.

### Effect of Ca^+2^ on the production and maturation of *Tth*PAC

**Figure 9:**
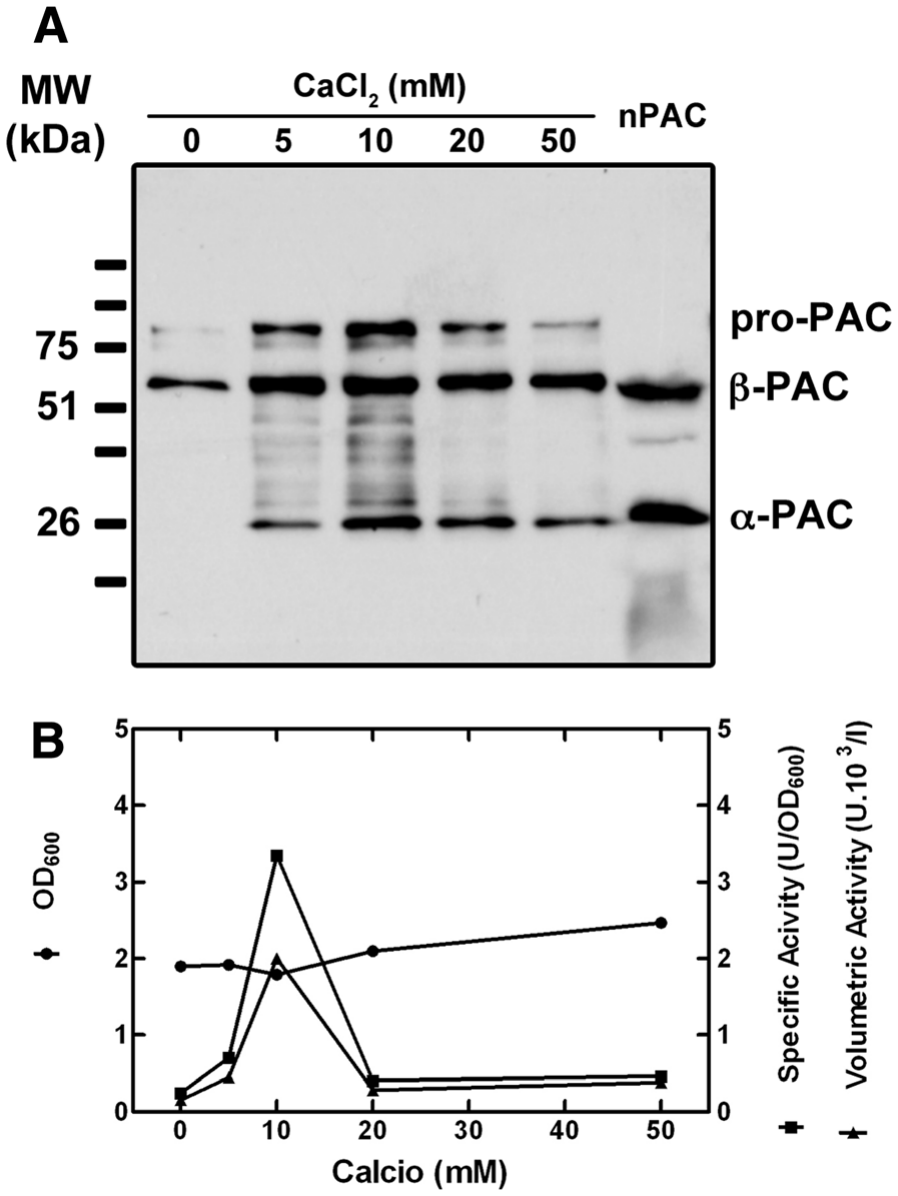
**Effect of calcium on* Tth *PAC overproduction in* E. coli *cells.** CaCl_2_ concentrations up to 50 mM were added to the LB media at the beginning of *E. coli* cells cultivation. *Tth*PAC maturation and activity were evaluated. (**A**) Western blot against α- and β-*Tth*PAC subunits. Lane 1–5, increasing calcium concentrations; lane 6 (C), *Tth*PAC matured in *Tth* cells. (**B**) Optical density at 600_nm_ (circles), specific (squares) and volumetric (triangles) activity of *Tth*PAC.

The crystal structure of *Eco*PGA (Protein Data Bank access number 1PNK) [27] and of the Bro1 mutant of *Providencia rettgeri* PGA [28], as well as analyses of induced coupled plasma-atomic emission spectroscopy on *Afae*PGA [29], revealed a tightly bound calcium ion in the structure of these three proteins. An amino acid sequence alignment of these PACs with the one from *Tth* showed that three of the six calcium co-ordinating residues identified in the above-mentioned PGAs are conserved in the *Tth*PAC sequence [Additional file 3. Calcium ions have been suggested to stabilize the PGA native state allowing its maturation to take place [30,31]. Indeed, the production of properly matured PGA proteins in *E. coli* cells has been improved in cultures supplemented with CaCl_2_[32,33]. Hence, the influence of Ca^+2^ ions on the expression and maturation of *Tth*PAC was tested. Increasing calcium concentrations up to 50 mM were added to LB media from the beginning of the cultivation. Cell density, *Tth*PAC expression and activity were analyzed, and results are summarized in Figure 9. While cell growth was slightly modified by the presence of Ca^+2^*Tth*PAC specific and volumetric activity was markedly improved at 10 mM Ca^+2^. Accordingly, western blot analysis of *Tth*PAC expression and maturation in *E. coli* cells showed increased signals for α- and β-subunits along with Ca^+2^ concentration of 10 mM (Figure 9). Regarding the auto-proteolytic processing of PAC, we observed that the size of both α- and β-subunits matched those of PAC obtained from *Tth* cells, suggesting a correct maturation of the pro-protein. Calcium proved to be a critical factor when producing functional *Tth*PAC, in the same way it was reported for the *Eco*PGA and *Afae*PGA [32,33]. The yield in active *Tth*PAC could be increased 13-fold when culture media was supplemented with 10 mM of CaCl_2_. From the results presented here we are able to suggest that calcium is part of the structure of *Tth*PAC as already described for some mesophilic PGAs [27-29].

### Purification of recombinant *Tth*PAC and evaluation of its maturation

**Figure 10:**
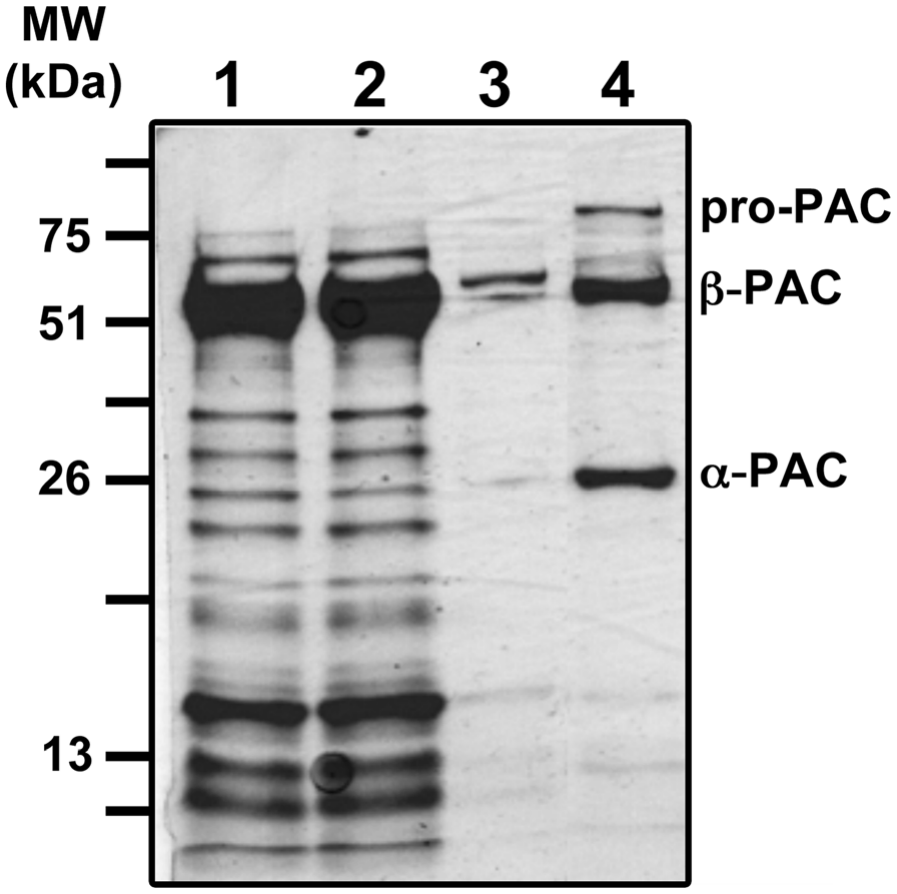
**HIS**_**6**_**::*Tth*PAC purification.** Fractions of total soluble protein (lane 1), protein flow through (lane 2), 5 mM imidazol protein elution (lane 3), and 150 mM imidazole protein elution were subjected to SDS-PAGE and stained with Coomasie Brillant Blue.

In order to confirm the correct maturation of *Tth* pro-PAC in *E. coli*, the recombinant protein was purified by IMAC chromatography (Figure 10). SDS-PAGE of the purified protein revealed two subunits of *Tth*PAC with relative molecular masses (M*r*) of 26.4 ± 1.0 kDa (α-subunit) and 54.3 ± 1.0 kDa (β-subunit) (Figure 10). MALDI-TOF and LC-MS/MS analyses were performed on each subunit. As expected, the N-terminal residue of the β-subunit corresponded to the catalytic Ser256. The C-terminus of the β-subunit was evident from the stop codon, whereas, the C-terminus of the α-subunit depends on the length of the linker peptide cleaved from the pro-*Tth*PAC. Since the α-subunit was mapped up to residue Arg246, then the auto-processing maturation must have eliminated a 9-amino acid spacer-peptide [Additional file 3. This is clearly a shorter linker peptide when compared to the 54- or 37-amino acid spacers of the mesophilic *Eco*PGA and *Afae*PGA, respectively [8,9]. However, since loops are likely the first structures that unfold during thermal denaturation, it is not surprising for a thermozyme to enhance its thermal stability through the shortening of loops [34]. Regarding the *Tth*PAC α-subunit, some disagreement between its molecular weight was experienced when producing *Tth*PAC in *Tth* or *E. coli* cells. As shown in Figures 3,4*Tth*PAC α-subunit presented a M*r* of aprox. 22 kDa while the same subunit (with a 20-amino acid His-tag replacing the 32-residue signal peptide) processed in *E. coli* cells was aprox. 26 kDa (Figures 8,9,10). *Tth* proteases, as most proteases from extremophilic bacteria, are serine proteases that are stable at high temperatures even in the presence of high concentrations of detergents and denaturing agents [35,36]. During cell lysis these proteases are normally activated, being probably responsible for the reduced size of the α-subunit observed in *Tth* cells. Since *Tth*PAC has a catalytic serine it is not possible to use serine-protease inhibitors during our protein purification protocols. Thus, the actual molecular weight of the α-subunit cannot be deduced from the α-PAC processed in *E. coli* nor from the one matured in *Tth* cells.

### Enzymatic characterization of *Tth*PAC

**Figure 11:**
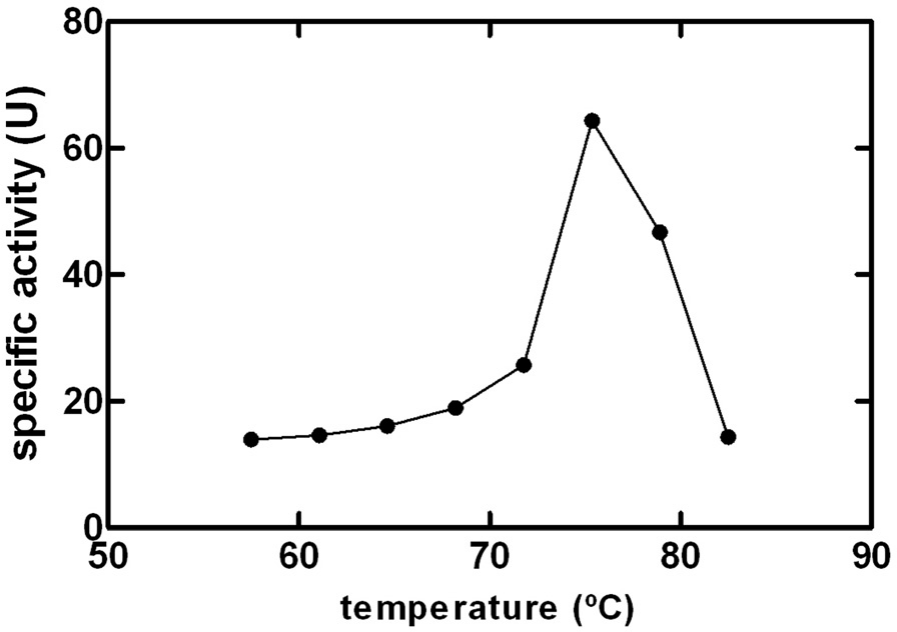
**Optimum reaction temperature of HIS**_**6**_**::*Tth*PAC.** The *Tth*PAC enzymatic activity was assayed in 20 mM MES pH 5.5, in the presence of 0.5 mM PenK and in a temperature range from 57.5 to 82.5°C.

Recombinant *Tth*PAC purified from *E. coli* cells was characterized biochemically. To examine its substrate selectivity, several aromatic and aliphatic penicillins, cephalosporins and homoserine-lactones were assayed for deacylating activity and results are summarized in Table 1. *Tth*PAC was active only on penicillins. Penicillin K (octanoyl-penicillin) was the best substrate, with the highest specificity constant value (16.12 mM^-1^.seg^-1^) compared to the other penicillins tested. *Tth*PAC activity was assayed at different pH values. In contrast to what is usually described for PAC enzymes we observed an optimal pH of 4.0 [Additional file 4. Also, the *Tth*PAC enzymatic activity was assayed in a range from 57.5 to 82.5°C. The highest hydrolytic activity of *Tth*PAC was achieved at 75°C (Figure 11). The preference for hydrophobic acyl-chain penicillins has been described previously for the PAC of the actinomycete *Streptomyces lavendulae* and the aculeacin A acylase from *Actinoplanes utahensis*[37,38] (see Additional file 5 for a straightforward comparison). In fact, a new subfamily of penicillin K acylases (PKAs) has been proposed to classify these two enzymes [38]. Clearly, from the results presented here, *Tth*PAC should be included in this subfamily and thus be renamed *Tth*PKA. Another common feature between *A. utahensis* PKA and *Tth*PKA is that they are both thermostable enzymes with an optimum reaction temperature of aprox. 75°C. However, while the half-life of the *A. utahensis* acylase at 65°C was calculated to be 477 min [38], *Tth*PAC exhibited a half-life of 552 min. (9.2 h) at 75°C [Additional file 6.

**Table 1:** **Kinetic parameters of** ***Tth*** **PAC for the hydrolysis of different substrates**

**Substrate**	**R (acyl chain)**	**K**_**m**_**(mM)**	**k**_**cat**_	**k**_**cat**_**/K**_**m**_
Penicillin K	Octanoyl	0.32 ± 0.05	5.16 ± 0.13	16.12
Penicillin dihydro F	Hexanoyl	0.98 ± 0.22	1.46 ± 0.06	1.49
Penicillin F	hexenoyl	0.94 ± 0.18	0.90 ± 0.03	0.96
Penicillin V	Phenoxyacetyl	0.74 ± 0.11	0.57 ± 0.01	0.77
Penicillin G	Phenylacetyl	Approx. 2.90	Approx. 4.10^-4^	Approx. 1.10^-4^
Glutaryl-7-ACA	Glutaryl	nd	nd	nd
Cephalosporin C	7-aminoadipyl	nd	nd	nd
C4-HSL	Butiryl	nd	nd	nd
C8-HSL	Octanoyl	nd	nd	nd

nd: no detectable activity.

While the actinomycetal PKAs share 40% identity, *Tth*PAC has only between 16-18% of identical residues with the former proteins. However when comparing the amino acid sequence of these three proteins with the ones of PACs with aromatic active centers (such as *Eco*PGA, *Afae*PGA, *P. rettgeri* PGA), some replacements that could explain the preference for an aliphatic or aromatic penicillin become evident. Two of the Phe identified as stabilizing residues of the phenylacetyl moiety of penicillin G in *Eco*PGA (α172 and β57) [27], are replaced by Ser/Gly and Ile, respectively, in PKAs [Additional file 3.

## Conclusions

A PAC enzyme from the extreme thermophile *Tth* HB27 strain was identified in the outer side of the cytoplasmic membrane. This is the first reported PAC from a extreme thermophile and owing to its potential use as a thermostable biocatalyst, an N-terminal 32-amino acid deletion version of *Tth*PAC was successfully overexpressed in *E. coli* cells using chaperone co-expression and calcium supplementation of the culture medium in order to enhance productivity and activity. The recombinant protein was purified and the maturation of the precursor in the cytoplasm of *E. coli* leading to an active enzyme was confirmed by MALDI-TOF analysis of the individual subunits. In the light of these data, a similar maturation process and mechanistic features as EcoPAC (N-terminal nucleophile Ser, structural calcium, activity-modulating linker peptide) can be hypothesized for this enzyme. Enzymatic characterization of *Tth*PAC was performed and its preference for aliphatic-chain penicillins was established, in good correlation with the aromatic for aliphatic substitutions found in multiple alignments for well-characterized. Industrial preparations of PenG obtained as bulk materials by fermentation contain up to 3% aliphatic penicillins, which cannot be hydrolyzed by PenG acylases and represent a significant amount in large-scale operations [37]. These “impurities” can now be hydrolyzed by PKAs, such as the one described in this work. Furthermore, penicillin acylases are not limited to penicillin hydrolysis and have a wide substrate specificity finding uses in other biocatalytic reactions, such as amide and ester formation under kinetic control or kinetic resolutions of amides [39]. *Tth*PAC showed an optimum reaction temperature of 75°C and thermal stability assays confirm that this enzyme clearly surpasses the stability of all other reported (native) acylases up to date, making it an interesting addition to the existing biocatalytic toolbox.

## Methods

### Materials and bacterial strains

All reagents used were of analytical grade and were purchased from Sigma (st. Louis, MO, USA) or Merck (Darmstadt, Germany). Penicillin G, V, K, F and dihydroF were kindly provided by Antibioticos S.A. (León, Spain). *Tth* Δ*pac* is a knockout in the *pac* gene (NCBI accession number TTC1972) derived from *Tth* HB27 strain. *Tth* Δ*slpA* is a knockout in the s*lpA* gene derived from *Tth* NAR1 strain [13]. *E. coli* DH5α [F– Φ80lacZΔM15 Δ(lacZYA-argF) U169 recA1 endA1 hsdR17 (rK–, mK+) phoA supE44 λ– thi-1 gyrA96 relA1] was used for subcloning steps and *E. coli* BL21(DE3) [F– ompT hsdSB(rB–, mB–) gal dcm (DE3)] was used as the host for recombinant protein expression.

### Plasmid constructions

All the primers used in this work are listed in [Additional file 7: Table S2]. In order to produce antibodies against the α- and β-subunits of PAC, each subunit was amplified from *Tth* HB27 genome using the primers αNdeIFw and αEcoRv for the α-subunit and βNdeIFw and βEcoRv for the β-subunit. The DNA fragments were cloned into the pET28b + plasmid between the *Nde*I and *EcoR*I restriction sites, yielding plasmids pET28αPAC and pET28βPAC. For the expression of the fusions with sGFP [12], *Tthpac* leader sequence, the entire *Tthpac* gene, *Tthpac* devoid of its leader sequence and *Tth* β-glycosidase codifying gene (NCBI accession number TTP0042) were amplified from *Tth* HB27 genome using the pairs of primers lsFw/lsRv, pacFw/pacRv, ΔSppacFw /pacRv, and βglyFw/βglyRv. The gene fusions were cloned into the plasmid pMKPnqosGFP [4]. For the expression of the chimeric protein Sp_*Eco*_-PAC_*Tth*_, the codifying sequence for the signal peptide of *E. coli* PGA was amplified with the primers EcoFw and EcoRv. The *Tth pac* gene devoid of its leader sequence was amplified from *Tth* HB27 genome with the primers ΔSppacFw and ΔSppacRv. The gene fusion was assembled by overlap extension PCR and ligated to the pMKPnqo expression plasmid. For the homologous expression of *Tth*PAC, the *pac* gene was amplified from *Tth* HB27 genome with the primers pac2Fw and pac2Rv. The gene was ligated to the pWUR112/77-1 [21] expression plasmid and the construction was named pWURPAC. For the heterologous expression of *Tthpac*, the ΔSppac2Fw primer and the ΔSppac2Rv primer were used in a PCR reaction for the amplification of a 2223 bp fragment corresponding to the *Tthpac* gene without the 5´ 96-nucleotide codifying sequence. The amplified gene was cloned into the pET28a plasmid between the *Nde*I and *EcoR*I restriction sites, yielding plasmid pET28a-ΔSpPAC. The construction was then transformed into *E. coli* DH5α competent cells. Plasmids were extracted and their inserts were sequenced to confirm the absence of mutations in *Tthpac* gene.

### Confocal microscopy

Fluorescence microscopy was performed using a Zeiss Inverted LSM510 confocal microscope. Z-stacks were obtained using a Zeiss 100x/1.3 oil Plan-Neofluar objective lens and parameters appropriate to comply with the Nyquist criteria for image sampling. Images were subjected to linear deconvolution using the Huygens System 2.2 software (Scientific Volume Imaging B.V., Hilversum. the Netherlands). Adobe Photoshop and Image J (Wayne Rasband, NIH, USA) were used for final assembly of the images. For sGFP fusion experiments, *Tth* HB27 strain was transformed with each one of the sGFP fusions (β-glycosidase-sGFP, ΔSp*Tth*PAC-sGFP, *Tth*PAC-sGFP and Sp_TthPAC_-sGFP) and was grown aerobically in TB liquid media (Ramirez-Arcos et al., 1998) at 70°C with mild shaking (150 rpm) up to an OD_550_ of 0.2-0.4 before cell harvesting by low-speed centrifugation (3,000 *x*g, 5 min). Mowiol was added after washing the cells with Milli-Q water.

### Isolation of the membrane fraction of *Tth* cells

Bacteria in mid-log growth phase were harvested by centrifugation at 5000 *x*g for 5 min at room temperature. The pellet was washed in TE 1X (Tris 10 mM pH 8, EDTA 1 mM) buffer and resuspended in TE. Cells were disrupted in a LABSONIC U sonicator (B. Braun) (2 times 30 seconds – 1 minute). Intact cells were removed by centrifugation at 5000 *x*g for 5 min. The supernatant was ultracentrifuged 30 min at 201,240 *x*g at 4°C in a Beckman TL-100. The pellet was washed in 10 mM Tris–HCl pH 7.5 and was centrifuged again in the same conditions. The final pellet corresponded to the membrane fraction.

### Selective solubilization of membrane components

Selective solubilization of the cytoplasmic membrane by non-ionic or weakly-ionic detergents is a widely used method to separate the inner and outer membrane components in Gram negative bacteria [14-17]. The membrane fraction was resuspended in 10 mM Tris–HCl pH 7.5 and 1% Triton X-100 or 1% Sarkosyl and incubated for 30 min at 37°C. Samples were then ultracentrifuged 30 min at 201,240 *x*g and 4°C. Supernatant was stored and the pellet was washed once again in the same buffer and centrifuged in the same conditions. Both fractions were subjected to SDS-PAGE and immunodetection of *Tth*PAC subunits.

### Trypsin accessibility

*Tth* NAR1 Δ*slpA* strain was grown at 70°C under aerobic conditions up to saturation. The bacteria were harvested by centrifugation at 5000 *x*g for 5 min at room temperature, washed once with 10 mM Tris–HCl pH 8.0, and the resulting pellet was resuspended in the same buffer up to a final cell concentration of 10^10^ cells/ml. Trypsin treatment was carried out using two different concentrations of enzyme at 37°C in the presence or absence of 5 or 10 mM EDTA. *Tth*PAC integrity was analyzed by western blot.

### Homologous expression

The *Tth* HB27 Δ*pac* mutant strain was transformed with the plasmid pWUR112/77-1 [21] carring the *pac* gene (pWURPAC) and was plated onto TB (Ramírez-Arcos *et al*., 1998) agar with bleomycin [21]. The constitutive expression of PAC was carried out growing the clones aerobically in TB liquid media (Ramírez-Arcos *et al*., 1998) with bleomycin at 70°C and 150 rpm until saturation. Cells were harvested by centrifugation at 4,000 *×* g and 4°C for 15 min. Cell pellets were resuspended in 50 mM Tris buffer pH 7.5 and 50 mM NaCl and disrupted by sonication on ice bath (2 rounds of 45 secs), using a sonicator LABSONIC U (B. Braun).

### Heterologous expression and purification of PAC

*E. coli* BL21(DE3) strain was transformed with the plasmid pET28a carring the ΔSp-*Tthpac* gene (pET28a-ΔspPAC) and was plated on LB agar with kanamycin (30 μg mL-1). The strain *E. coli* BL21(DE3) pET28a-ΔspPAC was then transformed with the vectors pGTf2, pTf16, pGro7 or pKJE7 (Takara Bio Inc), carrying the genes for GroEL-GroES, Trigger factor, GrpE and DnaK-DnaJ chaperone systems. Transformants were plated onto LB agar containing kanamycin (30 μg mL-1) and chloramphenicol (30 μg mL-1). Both transformations were based on the calcium temperature shock method.

The inoculums for batch protein production were prepared by overnight cultivation of the selected clone in 100 mL shake flaks with 20 ml of LB medium at 37°C. For protein production 1.5 mL of the corresponding inoculum culture was transferred to 150 mL of fresh LB medium supplemented with 10 mM CaCl_2_, and containing 0.5 g.L^-1^ of L-arabinose for chaperone induction. Cells were cultivated at 37°C and 200 rpm until they reached the *Tth*PAC over-expression induction point, corresponding to an OD_600_ ≈ 0.6 ± 0.5. Induction was performed by addition of IPTG (isopropyl-β-D thiogalactopyranoside) to a final concentration of 1 mM, and cultivation was continued for 17 hours at 22°C and 200 rpm. Cells were harvested by centrifugation (4,000 *×* g for 15 min) at 4°C. Cell pellets were resuspended in 10 mL of lysis buffer (50 mM sodium phosphate pH 7.0, 300 mM NaCl) and disrupted by sonication on ice bath (3 rounds of 10 min, 0.6 pulse and 40% power), using a sonicator LABSONIC U (B. Braun). Soluble and insoluble protein fractions were separated by centrifugation at 12,000 *×* g for 15 min at 4°C. The supernatant containing the His-tagged protein was partially purified from thermolabile *E. coli* proteins by a heat-shock treatment of 20 min at 65°C. Thermostable proteins were recovered from the supernatant after centrifugation (12,000 *×* g for 30 min) at 4°C, and subjected to IMAC chromatography using a Talon Cell-through resin (BD Biosciences) previously equilibrated with lysis buffer. The column was washed once with 1 volume of the lysis buffer. The His-tagged protein was eluted with 150 mM imidazole, diafiltrated and concentrated with Amicon Ultra-15 10 kDa centrifugal devices ((Millipore) using 50 mM sodium phosphate pH 7.0, 5 mM NaCl, 0.5 mM CaCl_2_, and stored at −20°C until use. The use of stabilizing additives like polyols was avoided because we observed interference with components of the reaction mixture. Protein content was determined using the Bio-Rad protein assay dye reagent concentrate (Bio-Rad, USA) with bovine serum albumin as standard. Samples for SDS-PAGE separation were prepared in 5× SDS-PAGE loading buffer and heated for 5 min at 95°C. Electrophoresis of protein samples was done with 12% (w/v) SDS-PAGE and the gel was stained with Coomassie Brilliant Blue R-250. Identical samples were electroblotted on PVDF membranes and incubated with polyclonal rabbit antibodies against the α- and β-subunit of *Tth*PAC, and later with an alkaline phosphatase-conjugated goat anti-rabbit antibody.

### MALDI-TOF/MS analysis

Metal affinity purified His_6_::ΔSp*Tth*PAC was subjected to SDS-PAGE on 12% polyacrylamide gels and the proteins were stained with Coomassie Brillant Blue R-250 solution. The polypeptide bands corresponding to α- and β-subunits of *Tth*PAC protein were excised from the gel and subjected to MALDI-TOF mass spectrometer analysis. The peptide identification by LC/MS/MS analysis was carried out in the ‘CBMSO Protein Chemistry Facility’, a member of ProteoRed network.

### Determination of *Tth*PAC enzymatic activity and thermal stability

Fluorescamine was used to follow the kinetics of *Tth*PAC against penicillins, cephalosporins or homoserine-lactones, through the reaction with the primary amine group of the corresponding reaction products. Aliquots of 40 microliter of reaction mixture were taken at regular intervals and were immediately frozen in dry ice after which 140 μl of 200 mM acetate buffer pH 4.5 and 20 μl of 1 mg.mL^-1^ fluorescamine in acetone were added. After a 60-min incubation at room temperature fluorescence was determined (Exc. 380 nm - Em. 530 nm) in a FLUOStar OPTIMA plate reader (BMG LabTech). All experiments were performed in duplicate, and the effect of non-enzymatic hydrolysis of substrates was subtracted. Substrate selectivity was analyzed using penicillin K, F, DHF, V or G, glutaryl-7 amino cephalosporanic acid (glutaryl-7ACA), cephalosporine C, and butyryl- or octanoyl-homoserine lactones (C4- or C8-HSL, respectively). Kinetic parameters *V*_max_ and *K*_m_ were determined by measuring the initial rate of hydrolysis on a range of substrate concentrations from 0.1 to 50 mM, using 0.8-2.0 μg of purified enzyme in 20 mM MES pH 5.5 for up to 1 h at 65°C. Optimum pH for the hydrolysis of 2.5 mM penicillin K was assayed at 65°C in 20 mM Britton-Robinson buffer at pH 3, 4, 5, 6, 7, 8 or 9 [40]. The temperature giving maximum penicillin K hydrolysis was determined by incubating 0.2 μg of enzyme with 0.5 mM of penicillin K in 20 mM MES pH 5.5, in a reaction volume of 50 μl at various temperatures within the range of 57.5°C to 82.5°C. The reaction was carried out in a 96-well PCR plate on a Thermal Cycler (Bio-Rad) running in gradient mode. After the reaction, the tubes were immediately chilled on ice. Thermal stability was determined incubating 0.08 mg/ml solutions of purified *Tth*PAC in 50 mM phosphate buffer pH 7.5 at 65 and 75°C and measuring the residual hydrolytic activity at regular intervals as detailed above using 5 mM penicillin K as substrate.

## Abbreviations

*Tth*, *Thermus themophilus*; PAC, penicillin acylase; PGA, penicillin G acylase; PKA, penicillin K acylase; 6-APA, 6-aminopenicillanic acid; *t*_1/2_, half-life; ORF, open reading frame; sGFP, superfolder green fluorescent protein; Sp, signal peptide; SlpA, S-layer protein; DrpA, nitrate respiration system regulatory protein; TAT, twin arginine transporter; TF, trigger factor.

## Competing interests

The authors declare financial competing interests. The contents of this manuscript have been partially patented by the authors’ institution (application number P201230729).

## Authors’ contributions

LLT carried out the cloning and the heterologous expression of ΔSp*Tth*PAC and Sp_*Eco*_-PAC_*Tth*_, the purification and the biochemical characterization of PAC protein, and drafted the manuscript. ACC participated in the heterologous expression of both proteins. EF performed the PAC subcellular location experiments, the trypsine accessibility assays, the PAC homologous expression and contributed to draft the manuscript. AH drafted the manuscript, and AH and JB critically revised and corrected the manuscript. All authors read and approved the final manuscript.

## Supplementary Material

Additional file 1 **Heterologous expression of the chimeric protein Sp**_***Eco***_**-PAC**_***Tth***_**in* E. coli *cells.***Tth*PAC α- and β-subunit were immunodetected in the cytoplasmic fraction (upper and middle panels) and in the periplasmic fraction (lower pannel) of *E. coli* cells from BL21 strain(1, A and G); Rosetta-gami2 strain (2, B and H); BL21 strain co-expressing GroEL/ES and trigger factor (3, C and I), trigger factor alone (4, D and J), GroEL/ES (5, E and K) or DnaK/J and GrpE (6, F and L). Protein samples were partially purified (+) or not (−) from *E. coli* thermolabile proteins by a 20 min-incubation at 65°C and later centrifugation. Click here for file

Additional file 2 **Chaperone elimination after co-expression with HIS**_**6**_**::* Tth *PAC.*** Tth *PAC was co-expressed with GroEL/ES in * E. coli * BL21 cells. Total soluble protein fraction was incubated in the absence (lanes 1–4) or in the presence of 5 mM ATP/10 mM Cl_2_Mg (lanes 5–8) [41] for 2 h at 4°C. In order to separate the * Tth *PAC from the detached GroEL/ES a 0-, 30-, 45- or 60-min incubation at 65°C and later centrifugation was performed. Immunodetection of β-*Tth*PAC shows that the mobility of this subunit (aprox. 60 kDa) is reduced when GroEL is eliminated (also 60 kDa). Click here for file

Additional file 3 **Sequence alignment of PKAs and characterized PGAs.** TthPKA, * Thermus thermophilus * HB27 PKA [TTC1972]; AutaPKA, *Actinoplanes utahensis* PKA [P29958]; SlavPKA, *Streptomyces lavendulae* PKA [AY611030]; EcoPGA, *Escherichia coli* PGA [P06875]; AfaePGA, *Alcaligenes faecalis* [ADD11517]; PretPGA, *Providencia rettgeri* PGA [AAP86197]; KcitPGA, *Kluyvera citrophila* [AAA25047]. Accession numbers to public databases are provided between brackets. *Tth*PAC α- and β-subunit extensions determined by MALDI-TOF analysis are shown with a red and blue arrow, respectively. Residues involved in calcium co-ordination are shown in yellow and identified with a letter “a” below the corresponding alignment column. Penicillin G-binding residues that differ within PGAs and PKAs are shown in red and blue color, respectively, and are identified with a letter “b” below the corresponding alignment column. Click here for file

Additional file 4 **Optimum pH of HIS**_**6**_**::*Tth*PAC.** The *Tth*PAC enzymatic activity was assayed at 65°C in the presence of 2.5 mM PenK and in 20 mM Britton-Robinson buffer at pH 3, 4, 5, 6, 7, 8 or 9. Click here for file

Additional file 5 **Comparison of the kinetic data of TthPAC with other penicillin acylases.** Data were measured at 40°C unless otherwise stated. Click here for file

Additional file 6 **Thermal inactivation course of TthPAC.** Solutions containing 0.08 mg/ml of purified *Tth*PAC were incubated in 50 mM phosphate buffer pH 7.5 at 65 and 75°C. The residual hydrolytic activity was determined using 5 mM penicillin K as substrate. Click here for file

Additional file 7 Table S2. Primers used in this work. Click here for file
