# Supplementary figures and images for: Functional expression of a penicillin acylase from the extreme thermophile Thermus thermophilus HB27 in Escherichia coli
Source: Microb Cell Fact. 2012 Aug 9;11:105. doi: 10.1186/1475-2859-11-105 (PMC3461476; doi:10.1186/1475-2859-11-105)

**
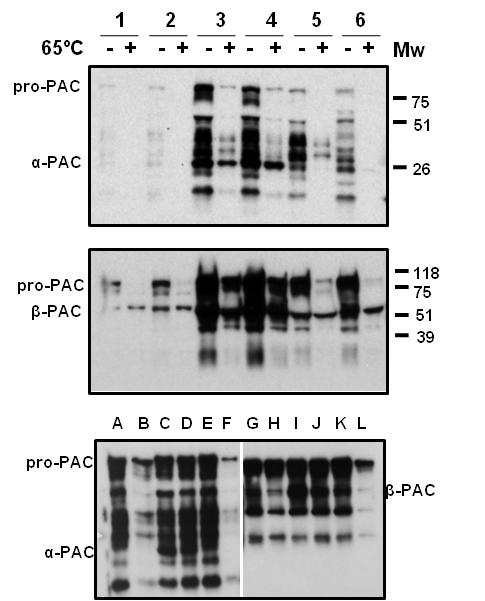
Additional file 1**

Supplement: Additional file 1 — Heterologous expression of the chimeric protein SpEco-PACTthin E. coli cells.TthPAC α- and β-subunit were immunodetected in the cytoplasmic fraction (upper and middle panels) and in the periplasmic fraction (lower pannel) of E. coli cells from BL21 strain(1, A and G); Rosetta-gami2 strain (2, B and H); BL21 strain co-expressing GroEL/ES and trigger factor (3, C and I), trigger factor alone (4, D and J), GroEL/ES (5, E and K) or DnaK/J and GrpE (6, F and L). Protein samples were partially purified (+) or not (−) from E. coli thermolabile proteins by a 20 min-incubation at 65°C and later centrifugation. [file 1475-2859-11-105-S1.docx]

**Additional file 2**

**
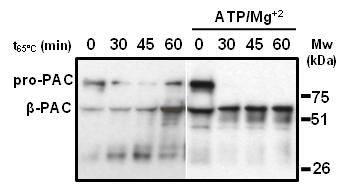
**

Supplement: Additional file 2 — Chaperone elimination after co-expression with HIS6:: Tth PAC. Tth PAC was co-expressed with GroEL/ES in E. coli BL21 cells. Total soluble protein fraction was incubated in the absence (lanes 1–4) or in the presence of 5 mM ATP/10 mM Cl2Mg (lanes 5–8) [41] for 2 h at 4°C. In order to separate the Tth PAC from the detached GroEL/ES a 0-, 30-, 45- or 60-min incubation at 65°C and later centrifugation was performed. Immunodetection of β-TthPAC shows that the mobility of this subunit (aprox. 60 kDa) is reduced when GroEL is eliminated (also 60 kDa). [file 1475-2859-11-105-S2.docx]

**Additional file 4**

**
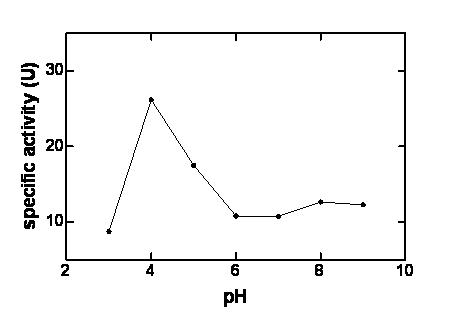
**

Supplement: Additional file 4 — Optimum pH of HIS6::TthPAC. The TthPAC enzymatic activity was assayed at 65°C in the presence of 2.5 mM PenK and in 20 mM Britton-Robinson buffer at pH 3, 4, 5, 6, 7, 8 or 9. [file 1475-2859-11-105-S4.docx]

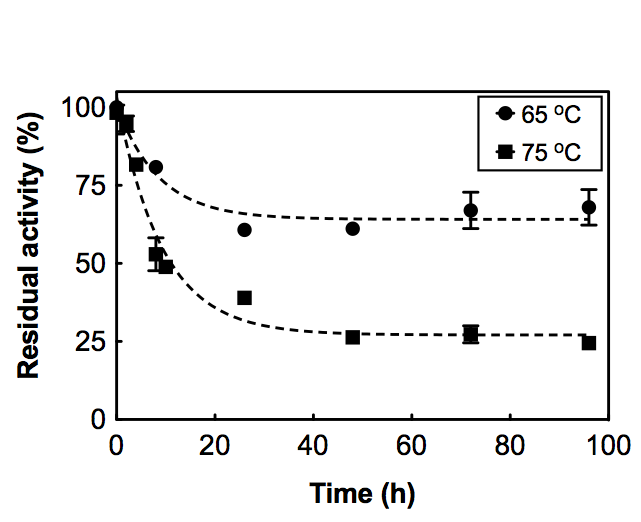

Supplement: Additional file 6 — Thermal inactivation course of TthPAC. Solutions containing 0.08 mg/ml of purified TthPAC were incubated in 50 mM phosphate buffer pH 7.5 at 65 and 75°C. The residual hydrolytic activity was determined using 5 mM penicillin K as substrate. [file 1475-2859-11-105-S6.png]
