# Supplementary material for: Functional expression of a penicillin acylase from the extreme thermophile Thermus thermophilus HB27 in Escherichia coli
Source: Microb Cell Fact. 2012 Aug 9;11:105. doi: 10.1186/1475-2859-11-105 (PMC3461476; doi:10.1186/1475-2859-11-105)
Supplement: Additional file 3 — Sequence alignment of PKAs and characterized PGAs. TthPKA, Thermus thermophilus HB27 PKA [TTC1972]; AutaPKA, Actinoplanes utahensis PKA [P29958]; SlavPKA, Streptomyces lavendulae PKA [AY611030]; EcoPGA, Escherichia coli PGA [P06875]; AfaePGA, Alcaligenes faecalis [ADD11517]; PretPGA, Providencia rettgeri PGA [AAP86197]; KcitPGA, Kluyvera citrophila [AAA25047]. Accession numbers to public databases are provided between brackets. TthPAC α- and β-subunit extensions determined by MALDI-TOF analysis are shown with a red and blue arrow, respectively. Residues involved in calcium co-ordination are shown in yellow and identified with a letter “a” below the corresponding alignment column. Penicillin G-binding residues that differ within PGAs and PKAs are shown in red and blue color, respectively, and are identified with a letter “b” below the corresponding alignment column. [file 1475-2859-11-105-S3.docx]

**Additional file 3**

* 20 * 40 * 60 * 80 * 100
TthPKA : MLFPTMKRFLRGLVWLLGLFLFLALLLGFSGYLYLRASLPQGEGRIALEGLSAPVEVVRDGKGVVRIRAATLKDLFFAQGFVHAQERLWQMEFQRRVGQGRLSEILGE- : 108
AutaPKA : ----------------MTSSYMRLKAAAIAFGVIVATAAVPSPASGREHDGGYAALIRRASYGVPHITADDFGSLGFGVGYVQAEDNICVIAESVVTANGERSRWFGAT : 93
SlavPKA : ----------MTFRNRLRLFAVSGLALFTVSASLPPAAASGAPEARHPSGGGLSATVRYTEYGIPHIVAKDYANLGFGTGWAQAADQVCTLADGFVTVRGERSKFFGPD : 99
EcoPGA : ----------------------MKNRNRMIVNCVTASLMYYWSLPALAEQSSSEIKIVRDEYGMPHIYANDTWHLFYGYGYVVAQDRLFQMEMARRSTQGTVAEVLGK- : 86
AfaePGA : ------------------------MQKGLVRTGLVAAGLILGWAGAPTHAQVQSVEVMRDSYGVPHVFADSHYGLYYGYGYAVAQDRLFQMDMARRSFVGTTAAVLGPG : 85
PretPGA : -------------------------MKKHLISIAIVLSLSSLSLSSFSQS--TQIKIERDNYGVPHIYANDTYSLFYGYGYAVAQDRLFQMEMAKRSTQGTVSEVFGK- : 81
KcitPGA : ----------------------MKNRNRMIVNGIVTSLICCSSLSALAASPPTEVKIVRDEYGMPHIYADDTYRLFYGYGYVVAQDRLFQMEMARRSTQGTVSEVLGK- : 86

* 120 * 140 * 160 * 180 * 200 * 2
TthPKA : --------------ATLPQDRFLRTWGFYR-AARAAYGK--------LYPEEKEAVDAYAAGVNAFLASG----APLPPEFRLLG--FRPEPWTGPDVLVWAKMMSYDL : 188
AutaPKA : G-------------PDDADVRTTSSTQAIDDRVAERLLEGPRDGVRAPCDDVRDQMRGFVAGYNHFLRRTG--VHRLTDPACRGKAWVRPLSEIDLWRTSWDSMVRAGS : 187
SlavPKA : AAPDFSLSSAAKNLSSDLYFRGVRDSGTVEKLLKVPAPAG-------PSRDAKESMRGFAAGYNAWLRQNR---DRITDPACRGASWVRPVTALDVAVRGFALAVLGGQ : 198
EcoPGA : --------------DFVKFDKDIRRNYWPD-AIRAQIAA--------LSPEDMSILQGYADGMNAWIDKVNTNPETLLPKQFNTFG-FTPKRWEPFDVAMIFVGTMANR : 171
AfaePGA : E-----------QDAYVKYDMQVRQNFTPA-SIQRQIAA--------LSKDERDIFRGYADGYNAYLEQVRRRPE-LLPKEYVDFD-FQPEPLTDFDVVMIWVGSMANR : 172
PretPGA : --------------DYISFDKEIRNNYWPD-SIHKQINQ--------LPSQEQDILRGYADGMNAWIKQINTKPDDLMPKQFIDYD-FLPSQWTSFDVAMIMVGTMANR : 166
KcitPGA : --------------AFVSFDKDIRQNYWPD-SIRAQIAS--------LSAEDKSILQGYADGMNAWIDKVNASPDKLLPQQFSTFG-FKPKHWEPFDVAMIFVGTMANR : 171

20 * 240 * 260 * 280 * 300 * 320
TthPKA : SGNWEEELKRHRLLARGVSPKRLLELKPPYPEDAP---------------------TVLRAEDLKLPLK-REEAPSALLR-------------------------M--- : 247
AutaPKA : GALLDGIVAATPPTAAGP--------------------------------------ASAPEAPDAAAIAAALDGTSAGIG----------------------------- : 229
SlavPKA : GRGIDGITAAQPPTAAPP-------------------------------------AAGVTPKEAAAAAQRLLSTQNADMG----------------------------- : 241
EcoPGA : FSDSTSEIDNLALLTALK-DKYGVSQGMAVFNQLKWLVNPSAPTTIAVQESNYPLKFNQQNSQTAALLP-RYDLPAPMLDRPAKGADGALLALTAGKNRETIAAQFAQG : 278
AfaePGA : FSDTNLEVTALAMRQSLE-KQHGPERGRALFDELLWINDTTAP-------TTVPAPAAEHKPQAQAGTQDLAHVSSPVLAT-------------------ELERQDKHW : 254
PretPGA : FSDMNSEIDNLALLTALK-DKYGEQLGVEFFNQINWLNNPNAPTTISSEEFTYSDSQKTKNISQLNQIS-DYRLTAPMFERTAKDTTGKVLALSSQENNALIAKQYEQS : 273
KcitPGA : FSDSTSEIDNLALLTAVK-DKYGNDEGMAVFNQLKWLVNPSAPTTIAARESSYPLKFDLQNTQTAALLVPRYDQPAPMLDRPAKGTDGALLAVTAIKNRETIAAQFANG : 279
 **b a**
 * 340 * 360 * 380 * 400 * 420 *
TthPKA : ---APPRFMEASNNWVVAGSRTETGKPFLANDPHLALQAPSLWFLMALEAPG-LRAIGATLPGLPGVVIGRNERIAWGVTNVGADVEDLYLLEEVEGRG--YRYKGRVV : 350
AutaPKA : -----------SNAYGLGAQATVNGSGMVLANPHFPWQGAERFYRMHLKVPGRYDVEGAALIGDPIIEIGHNRTVAWSHTVSTARRFVWHRLSLVPGDPTSYYVDGRPE : 327
SlavPKA : -----------SNAVAFRGSTTANGRGLLLGNPHYPWDGGRRFWQSQQTIPGELNVAGGSLLGSTTVSIGHNADVAWSHTVATGVTLNLHQLTLDPADPTVYLVDGKPQ : 339
EcoPGA : GANGLAGYPTTSNMWVIGKSKAQDAKAIMVNGPQFGWYAPAYTYGIGLHGAG-YDVTGNTPFAYPGLVFGHNGVISWGSTAGFGDDVDIFAERLSAEKPGYYLHNGKWV : 386
AfaePGA : GGRGPDFAPKASNLWSTRPERVQEGSTVLINGPQFGWYNPAYTYGIGLHGAG-FDVVGNTPFAYPIVLFGTNSEIAWGATAGPQDVVDIYQEKLNPSRADQYWFNNAWR : 362
PretPGA : GANGLAGYPTTSNVWLVGKTKASGAKAILLNGPQFGWFNPAYTYGIGLHGAG-FNIVGNTPFAYPAILFGHNGHVSWGSTAGFGDGVDIFAEQVSPEDPNSYLHQGQWK : 381
KcitPGA : -ANGLAGYPTTSNMWVIGKNKAQDAKAIMVNGPQFGWYAPAYTYGIGLHGAG-YDVTGNTPFAYPGLVFGHNGTISWGSTAGFGDDVDIFAEKLSAEKPGYYQHNGEWV : 386
  **b a aa**

440 * 460 * 480 * 500 * 520 * 540
TthPKA : PYGVREEVIRVKGGREEVLKVRETVYG-PVITDALEDPPKTPMALRWVSLDEEDH---ILMAFLGVNRAGNWEEFK--KALLPYSAPSQNFVYADVEGN----IGYIAP : 449
AutaPKA : RMRARTVTVQTGSG-PVSRTFHDTRYG---PVAVVPGTFDWTPATAYAITDVNAGNNRAFDGWLRMGQAKDVRALK-AVLDRHQFLPWVNVIAADARGEALYGDHSVVP : 431
SlavPKA : RMTQRTVAVPVKGAAPVTRTQWWTRYGPVVTSLGAALPLPWTASTAYALNDPNAVNLRSADTSLGFSKARSTAGIER-ALHRSQGLPWVNTIAADRSGNSFFSQSQVLP : 447
EcoPGA : KMLSREETITVKNGQAETFTVWRTVHG-NILQTDQTTQTAYAKSRAWDGKEV--A---SLLAWTHQMKAKNWQEWT--QQAAKQALTI-NWYYADVNGN----IGYVHT : 482
AfaePGA : TMEQRKERIQVRGQADREMTIWRTVHG-PVMQFDYDQGAAYSKKRSWDGYEV--Q---SLLAWLNVAKARNWTEFL--DQASKMAISI-NWYYADKHGN----IGYVSP : 458
PretPGA : KMLSRQETLNVKGEQPITFEIYRTVHG-NVVKRDKTTHTAYSKARAWDGKEL--T---SLMAWVKQGQAQNWQQWL--DQAQNQALTI-NWYYADKDGN----IGYVHT : 477
KcitPGA : KMLSRKETIAVKDGQPETFTVWRTLDG-NVIKTDTRTQTAYAKARAWAGKEV--A---SLLAWTHQMKAKNWPEWT--QQAAKQALTI-NWYYADVNGN----IGYVHT : 482

* 560 * 580 * 600 * 620 * 640 *
TthPKA : GKFPVRKEG-HTGMVPVPG-NGEWDWL-GYRRPE-EWPQAFNPARGYLVTANHKVTPKGFPY---ALTYDWAEPYRAERIEELLLAKEK---------LSLEDMKAIQQ : 542
AutaPKA : RVTGALAAACIPAPFQPLYASSGQAVLDGSRSDCALGADPDAAVPGILGPASLPVRFRDDYVTNSNDSHWLASPAAPLEGFPRILGNERTPRSLRTRLGLDQIQQRLAG : 540
SlavPKA : RITDELAARCSTPLGQATYPSAGLAVLDGSTSACALGSDRDAVQPGIFGPGRMPTLKNAPYVENSNDSAWLTNADRPLTGYERVFGTTATQRSIRT-RGAIEDVAAMAE : 555
EcoPGA : GAYPDRQSG-HDPRLPVPG-TGKWDWK-GLLPFE-MNPKVYNPQSGYIANWNNS--PQKDYPASDLFAFLWGGADRVTEIDRLLEQKPRLT----A-DQAWDVIRQTSR : 580
AfaePGA : AFLPQRPAD-QDIRVPAKG-DGSMEWL-GIKSFD-AIPKAYNPPQGYLVNWNNK----PAPDKTNTDTYYWTYGDRMNELVSQYQQKDLFS----V-QEIWEFNQKASY : 554
PretPGA : GHYPDRQIN-HDPRLPVSG-TGEWDWK-GIQPFA-NNPKVYNPKSGYIANWNNS--PAKNYPASDLFAFLWGSADRVKEIDNRIEAYDKLT----A-DDMWAILQQTSR : 575
KcitPGA : GAYPDRQPG-HDPRLPVP--DGKWDWK-GLLSFD-LNPKVYNPQSGYIANWNNS--PQKDYPASDLFAFLWGGADRVTEIDTILDKQPRFT----A-DQAWDVIRQTSL : 579
  **a a**

660 * 680 * 700 * 720 * 740 * 760
TthPKA : DQK------SLLYRDFRPVLELLTPLSEGARAWRDRLLAWDGTMAPGSE--------EALVFALWYTELTRLPQREVGEAYWDEPRY---------------------L : 616
AutaPKA : TDGLPGKGFTTARLWQVMFGNRMHGAELVRDDLVALCRRQP--TATASNGAIVDLTAACTALSRFDERADLDSRGAHLFTEFLAGGIR--------------------- : 626
SlavPKA : RGRL-----RVTDLERQQLANRAPTGDLVAADVAKWCAALPGGTAVGSSGTPVDVSAACPVLRRWDRSVDSDSRGALLFDRFWRKAAAVP---------------AAEL : 644
EcoPGA : QDLN-----LRLFLPTLQAATSGLTQSDPRRQLVETLTRWDGINLLNDDGKT-WQQPGSAILNVWLTSMLKRTVVAAVPMPFDKWYSASGYETTQ-DG-PTGSLNISVG : 681
AfaePGA : SDVN-----WRYFRPHLEKLAQQLPADDSSKAALTMLLAWDGMEQDQGGQN---AGPARVLFKTWLEEMYKQVLMPVVPESHRAMYSQTGFATQQG--PNPGSINLSMG : 653
PretPGA : VDLN-----HRLFTPFLTQATQGLPSNDNSVKLVSMLQQWDGINQLSSDGKH-YIHPGSAILDIWLKEMLKATLGQTVPAPFDKWYLASGYETTQ-EG-PTGSLNISTG : 676
KcitPGA : RDL------LRLFLPALKDATANLAENDPRRQLVDKLASWDGENLVNDDGKT-YQQPGSAILNAWLTSMLKRTVVAAVPAPFGKWYSASGYETTQ-DG-PTGSLNISVG : 679

* 780 * 800 * 820 * 840 * 860 *
TthPKA : LKALKEGDKNCDQPET--------EYRESCLDYAALALERALDRK--EALGARAWGEVHRARFPHAVLT---HTPLKRLSDREVAFGGDRYTVNVGPFDPETLAMGHGP : 712
AutaPKA : FADTFEVTDPVRTPAPFWN-TTDPRVRTALADACNGSPASPSTRSVGDIHTDSRGERRIPIHGGRGEAG-----TFNVITNPLVPG-VGYPQVVHGTSFVMAVELG--- : 725
SlavPKA : WKVPFDAADPVRTPRGLNTAAPGVGKALADTVTELKAAGIALNAPLGEHQFVVRNGKRIPVGGGTESLG-----IWNKIEPVWNPAAGGYTEVSAGSSYIQAVG----- : 743
EcoPGA : AKILYEAVQGDKSPIPQAVDLFAGKPQQEVVLAALEDTWETLSKRYGNNVSNWKTPAMALTFRANNFFGVPQAAAEETRHQAEYQNRGTENDMIVFSPTTSDR-----P : 785
AfaePGA : TKVLLRALVLEAHPDPKRVNVFGERSSQEIMHTALQNAQARLSQEQGAQMARWTMPTSVHRFSDKNFTGTPQTMPGNTFAFTGYQNRGTENNRVVFDAKG--------- : 753
PretPGA : AKLLYESLLEDKSPISQSIDLFSGQPQNDVIRKTLNTTYQKMIEKYGDNPANWQTPATALTFRENNFFGIPQALPQENFHQNEYHNRGTENDLIVFTEEG--------- : 776
KcitPGA : AKILYEALQGDKSPIPQAVDLFGGKPEQEVILAALDDAWQTLSKRYGNDVTGWKTPAMALTFRANNFFGVPQAAAKEARHQAEYQNRGTENDMIVFSPTSGNR-----P : 783

880 * 900 * 920 * 940
TthPKA : SYRQIVDLSDMEGSLFVHPMGQSGHFLSRHYADLLPLWQRGDYLPMRFGAPLGRTLLLEPLPSP----- : 776
AutaPKA : -----PHGPS---GRQILTYAQSTNPNSPWYADQTVLYSRKGWDTIKYTEAQIAADPNLRVYRVAQRGR : 786
SlavPKA : -----WDNSRCPVARTLLTYSQSSNPNSPHYSDQTRLFSGERWVTSRFCEKDIARSPQLKVVRVHERR- : 806
EcoPGA : --VLAWDVVAPGQSGFIAPDGTV----DKHYEDQLKMYENFGRKSLWLTKQDVEAHKESQEVLHVQR-- : 846
AfaePGA : --VEFCDAMPPGQSGFTDRNGVR----SPHYEDQLKLYENFECKTMDVTHADIRRNAQSSTMLLIQPQP : 816
PretPGA : --VSAWDVVAPGQSGFISPQGKP----SPHYQDQLSLYQQFGKKPLWLNSEDVAPYIESTETLIIER-- : 837
KcitPGA : --VLAWDVVAPGQSGFIAPDGKA----DKHYDDQLKMYESFGRKSLWLTPQDVDEHKESQEVLQVQR-- : 844
