# Supplementary material for: Functional expression of a penicillin acylase from the extreme thermophile Thermus thermophilus HB27 in Escherichia coli
Source: Microb Cell Fact. 2012 Aug 9;11:105. doi: 10.1186/1475-2859-11-105 (PMC3461476; doi:10.1186/1475-2859-11-105)
Supplement: Additional file 5 — Comparison of the kinetic data of TthPAC with other penicillin acylases. Data were measured at 40°C unless otherwise stated. [file 1475-2859-11-105-S5.docx]

|  | **TthPAC (65 ^o^C)** | | | ***E.coli* PAC^a^** | | | ***Alcaligenes faecalis* PAC^b^** | | |
| --- | --- | --- | --- | --- | --- | --- | --- | --- | --- |
| **Substrate** | **K_m_ (mM)** | **k_cat_ (s^-1^)** | **k_cat_/ K_m_ (mM^-1^s^-1^)** | **K_m_ (mM)** | **k_cat_ (s^-1^)** | **k_cat_/ K_m_ (mM^-1^s^-1^)** | **K_m_ (mM)** | **k_cat_ (s^-1^)** | **k_cat_/ K_m_ (mM^-1^s^-1^)** |
| Penicillin K | 0.32±0.05 | 5.16±0.13 | 16.12 |  |  |  |  |  |  |
| Penicillin dihydro F | 0.98±0.22 | 1.46±0.06 | 1.49 |  |  |  |  |  |  |
| Penicillin F | 0.94±0.18 | 0.90±0.03 | 0.96 |  |  |  |  |  |  |
| Penicillin V | 0.74±0.11 | 0.57±0.01 | 0.77 |  |  |  |  |  |  |
| Penicillin G | Approx. 2.90 | Approx. 4.10-4 | Approx. 1.10-4 | 0.007 | 42 | 6000 | 0.0042 | 54 | 12857 |

|  | ***Kluyvera citrophila* PAC^c^** | | | ***S. lavendulae* PAC^d^** | | | ***A. utahensis* aculeacin A acylase^e^**  **(45 ^o^C)** | | |
| --- | --- | --- | --- | --- | --- | --- | --- | --- | --- |
| **Substrate** | **K_m_ (mM)** | **k_cat_ (s^-1^)** | **k_cat_/ K_m_ (mM^-1^s^-1^)** | **K_m_ (mM)** | **k_cat_ (s^-1^)** | **k_cat_/ K_m_ (mM^-1^s^-1^)** | **K_m_ (mM)** | **k_cat_ (s^-1^)** | **k_cat_/ K_m_ (mM^-1^s^-1^)** |
| Penicillin K |  |  |  | 0.14 | 22.71 | 165.3 | 1 | 33.3 | 34.79 |
| Penicillin dihydro F |  |  |  | 0.69 | 15.19 | 21.85 | 5.6 | 4.2 | 0.75 |
| Penicillin F | 0.047 | 4.7 | 100 | 1.2 | 7.69 | 6.41 | 15.1 | 1.9 | 0.13 |
| Penicillin V |  |  |  | 2.05 | 60.25 | 38.88 | 15.4 | 70.3 | 4.55 |
| Penicillin G | 0.018 | 56.5 | 3139 | 60.20 | 3.40 | 0.075 | 155.8 | 2.2 | 0.01 |

a. Alkema, W. B. L. *et al.* Characterization of the β-lactam binding site of penicillin acylase of *Escherichia coli* by structural and site-directed mutagenesis studies. *Protein Eng.* **13**, 857–863 (2000).

b. Švedas, V., Guranda, D., van Langen, L., van Rantwijk, F. & Sheldon, R. Kinetic study of penicillin acylase from *Alcaligenes faecalis*. *FEBS Lett.* **417**, 414–418 (1997).

c. Prieto, I., Martín, J., Arche, R., Fernández, P. & Pérez-Aranda, A. Penicillin acylase mutants with altered site-directed activity from *Kluyvera citrophila*. *Appl. Microbiol. Biotechnol.* **33**, 553–559 (1990).

d. Torres-Guzmán, R. *et al.* Substrate Specificity of Penicillin Acylase from *Streptomyces lavendulae*. *Biochem. Biophys. Res. Commun.* **291**, 593–597 (2002).

e. Torres-Bacete, J. *et al.* Newly discovered penicillin acylase activity of aculeacin A acylase from *Actinoplanes utahensis*. *Appl. Environ. Microbiol.* **73**, 5378–5381 (2007).
