# Supplementary material for: Functional expression of a penicillin acylase from the extreme thermophile Thermus thermophilus HB27 in Escherichia coli
Source: Microb Cell Fact. 2012 Aug 9;11:105. doi: 10.1186/1475-2859-11-105 (PMC3461476; doi:10.1186/1475-2859-11-105)
Supplement: Additional file 7 — Table S2. Primers used in this work. [file 1475-2859-11-105-S7.doc]

| **Name** | **Primer sequence (5´- 3´)** | **Restriction sites** (underlined in primer sequence) |
| --- | --- | --- |
| αNdeIFw | CGCCATATGCTGGAGGGGCTTTCC | *Nde*I |
| αEcoRv | AAAGAATTCTTAGGTGGGGGCGTCCT | *Eco*RI |
| βNdeIFw | AAAACATATGAGCAACAACTGGGTGGTG | *Nde*I |
| βEcoRv | AAAGAATTCTTAAGGCGAAGGAAGGGG | *Eco*RI |
| lsFw | AAAACATATGCTTTTTCCAACCATGA | *Nde*I |
| lsRv | AAAAGAATTCCAGGGCGATGCGGCCT | *Eco*RI |
| pacFw | CGCCATATGCTGGAGGGGCTTTCC | *Nde*I |
| pacRv | GAGGGAATTCAGGCGAAGGAAGGGGCT | *Eco*RI |
| βglyFw | AAACATATGACCGAGAACGCCGA | *Nde*I |
| βglyRv | AAAAGAATTCGGTCTGGGCCCGCG | *Eco*RI |
| EcoFw | CGACGCCATATGAAAAATAGAAATCGTATGATC | *Nde*I |
| EcoRv | GGCAGGGAAGCCAGTGCAGGTAAG | - |
| ΔSppacFw | TGCACTGGCTTCCCTGCCCCAAG | - |
| ΔSppacRv | AAAGAATTCTTAAGGCGAAGGAAGGGG | *Eco*RI |
| pac2Fw | AAAACATATGCTTTTTCCAACCATGA | *Nde*I |
| pac2Rv | AAAAGAATTCTTAAGGCGAAGGAAGGG | *Eco*RI |
| ΔSppac2Fw | AATTCCATATGTCCCTGCCCCAAGG | *Nde*I |
| ΔSppac2Rv | AAAGAATTCTTAAGGCGAAGGAAGGGG | *Eco*RI |

**Additional file 5. Table 2. Primers used in this work**
